# Electrospun Biomaterials in the Treatment and Prevention of Scars in Skin Wound Healing

**DOI:** 10.3389/fbioe.2020.00481

**Published:** 2020-06-03

**Authors:** Eoghan J. Mulholland

**Affiliations:** Gastrointestinal Stem Cell Biology Laboratory, Wellcome Trust Centre for Human Genetics, University of Oxford, Oxford, United Kingdom

**Keywords:** nanofibers, nanotechnology, electrospinning, polymer, drug delivery, tissue engineering, wound healing, scars

## Abstract

Electrospinning is a promising method for the rapid and cost-effective production of nanofibers from a wide variety of polymers given the high surface area morphology of these nanofibers, they make excellent wound dressings, and so have significant potential in the prevention and treatment of scars. Wound healing and the resulting scar formation are exceptionally well-characterized on a molecular and cellular level. Despite this, novel effective anti-scarring treatments which exploit this knowledge are still clinically absent. As the process of electrospinning can produce fibers from a variety of polymers, the treatment avenues for scars are vast, with therapeutic potential in choice of polymers, drug incorporation, and cell-seeded scaffolds. It is essential to show the new advances in this field; thus, this review will investigate the molecular processes of wound healing and scar tissue formation, the process of electrospinning, and examine how electrospun biomaterials can be utilized and adapted to wound repair in the hope of reducing scar tissue formation and conferring an enhanced tensile strength of the skin. Future directions of the research will explore potential novel electrospun treatments, such as gene therapies, as targets for enhanced tissue repair applications. With this class of biomaterial gaining such momentum and having such promise, it is necessary to refine our understanding of its process to be able to combine this technology with cutting-edge therapies to relieve the burden scars place on world healthcare systems.

## Introduction

Pathological scar formation is the physiological conclusion of wound healing, and so it is important to understand its underlying cellular and molecular processes in order to apprehend how a scar is formed, but also for the exploration of potential therapeutic targets. Currently, scarring is a huge burden on world healthcare, and the global scar treatment market is projected to represent as much as $34.9 billion by the year 2023^1^. Indeed, scarring can lead to many adverse side effects such as reduced mobility, compromised function in organs such as the liver or kidney, and the development of functional disabilities such as the psychological stress (Krafts, 2010; Sarrazy et al., 2011). A plethora of treatment options are available for scarring including topical treatments and dressings but are met with many limitations and are proving ineffective. This review will explore the use of electrospun nanofibers as novel instruments for efficient wound healing and reducing scar formation. The large surface area to volume ratio make electrospun fibers attractive options as they offer therapeutic incorporation capabilities whilst also being absorptive. A key focus of this review will be how these nanofibers can be applied alone, but also in conjunction with pharmacotherapies and cells for effective skin repair.

**Graphical Abstract:**
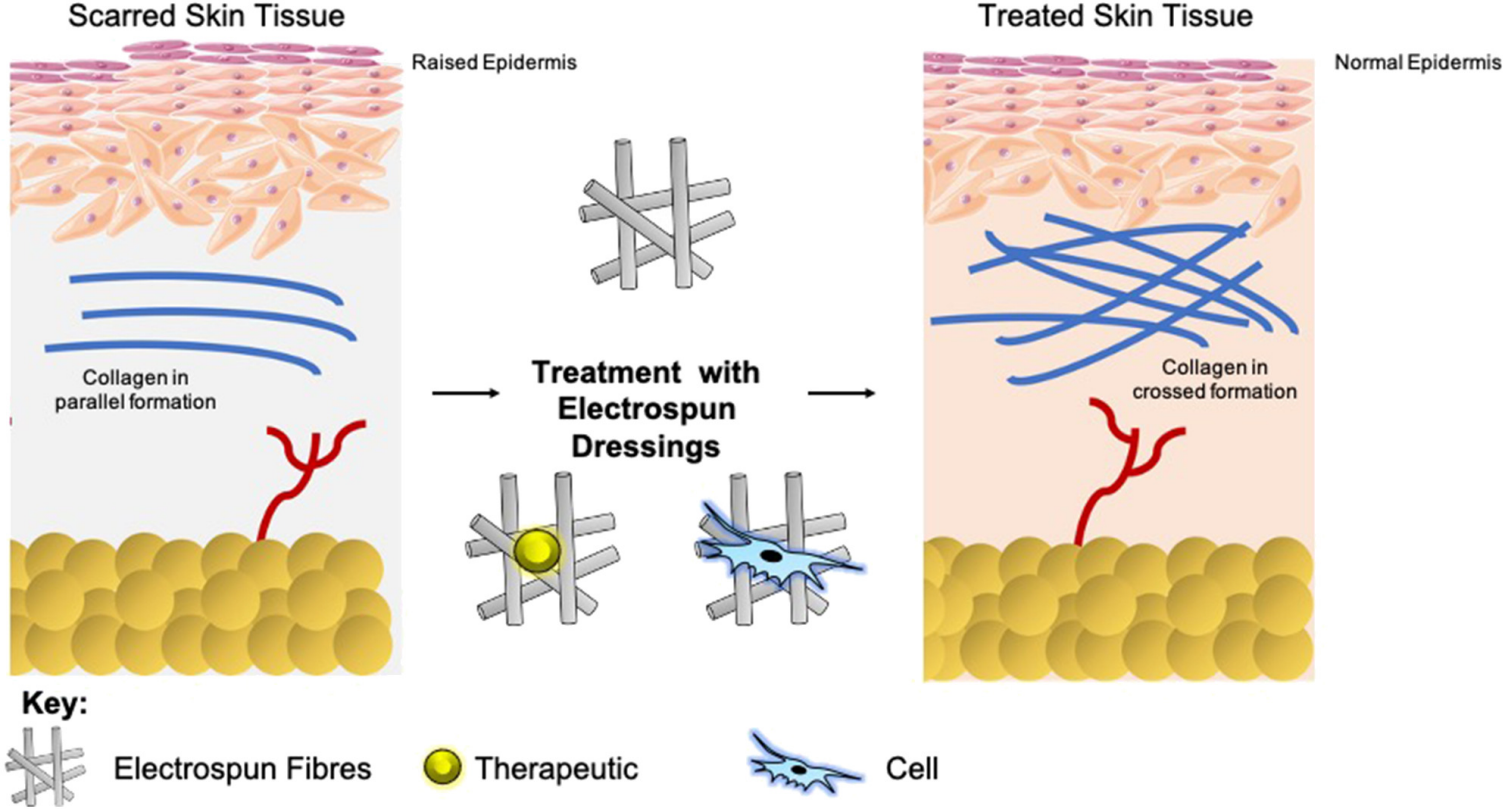


## Wound Healing

Wound healing is a highly complex process which stems from three well-defined phases: inflammation, proliferation, and remodeling. Inflammation is the immediate response phase, commencing with the contact of platelets from blood with exposed collagen at the site of injury (Qin, 2016). This contact initiates the formation of a fibrin clot, comprised of the platelets, thrombin, and fibronectin. The fibrin clot acts as a reservoir of cytokines and growth factors, stimulating inflammatory mediators to migrate to the wound, while also providing an architecture for infiltrating cells (Hsieh et al., 2017). For example, transforming growth factors (TGF-α, TGF-β) is a highly significant signaling pathway initiated by platelets within the fibrin clot (Ramirez et al., 2014). TGFs draw leukocytes to the injury site and initiate the inflammatory stage (Kryczka and Boncela, 2015). These leukocytes support the secretion of additional cytokines, e.g., platelet-derived growth factor (PDGF), interleukin-1 (IL-1), and fibroblast growth factor (FGF) (Grove and Kligman, 1983). During proliferation, the secondary stage of wound healing, TGF signaling becomes increasingly crucial, especially in cell types such as keratinocytes, macrophages, and fibroblasts, essential for the transcription of collagen, fibronectin, and proteoglycan. Additionally, TGFs prevent the release of protease enzymes responsible for the degradation of the matrix and activates inhibitors of protease production (Broughton et al., 2006). As proliferation progresses, fibroblasts are the principal cell type. Fibroblasts are of mesenchymal origin and are accountable for new matrix production, resulting in the restoration of tissue homoeostasis (Darby et al., 2014). Remodeling, the final stage, can last up to 1-year after injury. Collagenase enzymes secreted from fibroblasts, macrophages, and neutrophils, cleave the molecules of collagen, thereby breaking it down (Caley et al., 2015). This results in Type I collagen gradually replacing Type III collagen, which in time increases the tensile strength of the new tissue (Longaker et al., 2008). The collagen fibers in the wound tissue are thinner than that of normal dermal collagen. These thinner fibers will gradually thicken over time and organize along the stress lines of the injury. This resulting scar tissue, however, will never be as strong as the preceding normal tissue (White et al., 1971; Schilling, 1976; Corr et al., 2009). Many studies suggest that variations in inflammation during the wound healing process are directly related to the extent of scar tissue formation (Lim et al., 2006). For example, fetal wound healing presents with a lack of typical inflammatory markers and is “*scarless*” up to a certain age (Longaker et al., 2008). In adult wound healing, polymorphonuclear leukocytes are recruited to the site of injury, followed by macrophages and lymphocytes. Contrastingly, fetal wounds are void of polymorphonuclear leukocytes, and as healing progresses, fetal macrophages enter the wound site but in lesser numbers than that of an adult (Mackool et al., 1998). This characteristic lack of an inflammatory response may be credited to a dearth in appropriate signaling in fetal wounds, and the fundamentally immature condition of fetal inflammatory cell populations. Many non-healing wounds fail to switch from the inflammatory phase into the proliferative phase, thus resulting in abnormal wound repair.

## Scar Formation

Scars present as a significant burden to healthcare systems, and so are a catalyst for global research for prevention and reduction (Mirastschijski et al., 2015; Barnes et al., 2018). A mature scar consists predominantly of Type I collagen (Marshall et al., 2018). Within scar tissue, this collagen is arranged in bundles parallel to the skins surface, as opposed to a non-parallel conformation in normal skin. This parallel configuration in scar tissue equates to an overall reduction in tensile strength (van Zuijlen et al., 2003). The epidermal basement membrane presents with a more flattened nature as opposed to normal skin, as it does not contain the rete pegs which typically infiltrate the dermis (Monaco and Lawrence, 2003). Furthermore, scar tissue is void of other classic dermal adjuncts such as hair follicles or sweat glands (Fu et al., 2005; Kiani et al., 2018). Upon maturation, the concentration of fibroblasts with the scar tissue depletes, which in combination with the lack of dermal adjuncts results in a dermal layer comprising of few cells. The extracellular matrix (ECM) of this tissue has less elastin than healthy tissue, which contributes to the loss in tensile strength, and means that re-injury is more probable (Kordestani, 2019).

**Figure 1:**
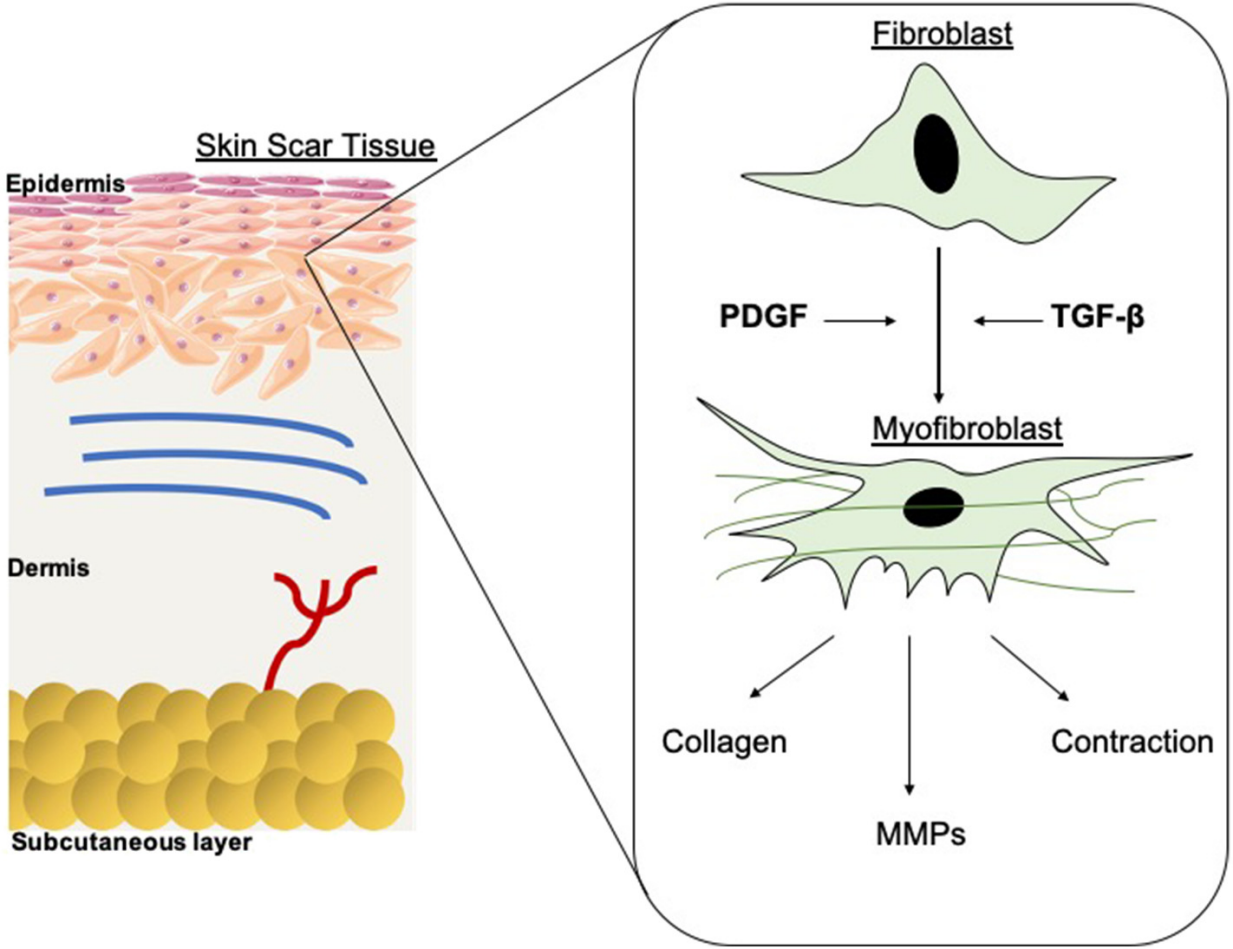
Schematic of fibroblast differentiation into myofibroblasts within scar tissue. TGF-β and PDGF stimulate the differentiation of myofibroblasts from fibroblasts, thus contributing to wound contraction. The contractile force is delivered by the myofibroblasts until re-epithelialisation is complete then they go through apoptosis. MMPs are also released and are essential in the remodeling phase of wound healing and scar formation. However, in hypertrophic scarring this myofibroblast depletion is not well-orchestrated, resulting in functional defects.

The extent of fibrosis post-injury varies between organs and tissues. When the molecular regulation of the remodeling phase of wound healing is inefficient or disturbed, more problematic scars occur: hypertrophic scars and keloids. Hypertrophic scars typically develop post-surgery or from other trauma such as burns (Carswell and Borger, 2019). Keloids contrast from hypertrophic scars in that they grow beyond the natural margins of the initial damaged tissue (Berman et al., 2017). These keloidal scars do not naturally revert, as opposed to hypertrophic scares which typically regress to a degree within 6 months. Histologically speaking, keloid, and hypertrophic scars are distinguishable by a difference in collagen fiber architecture, presence of myofibroblasts which are alpha-smooth muscle actin-positive, and the degree of angiogenesis (Carswell and Borger, 2019). Keloids are characterized by thick fibers of collagen, while hypertrophic scars encompass thin fibers organized in nodules. The dysregulation to normal collagen maturation is a central influencer on excessive scar formation. Hypertrophic scars contain high concentrations of microvessels, attributed to excess proliferation and loss of functionality of endothelial cells. This phenomenon can be traced back to myofibroblast hyperactivity and the resulting excess collagen fabrication. Myofibroblasts are the principal cell type responsible for scar contraction (Li and Wang, 2011), and are derived from fibroblasts ~2 weeks post-wounding (Singer and Clark, 1999). PDGF and TGF-β stimulate this cellular differentiation and the resultant contractile force exerted by the myofibroblasts enables wound edges in humans to come together, at a rate of ~0.75 mm a day (Figure 1) (Werner and Grose, 2003; Storch and Rice, 2005). Of course, in normal scars this wound contraction is an essential process; however, myofibroblasts typically go through apoptosis post-epithelialization, thus halting contractive pathways (Desmoulière et al., 2005). In hypertrophic scars, these myofibroblasts do not apoptose beyond epithelialization and so cause persistent contraction, resulting in functional implications to the skin (Ehrlich et al., 1994). Keloid scars, however, are smooth muscle actin negative (Ehrlich et al., 1994). This can be attributed to the presence of protomyofibroblasts in keloids, which can manufacture large quantities of ECM but not the force to contract lesions. This explains why functional defects resulting from contraction are only observed in hypertrophic scarring. Typically, the granulation tissue continues to expand and secrete growth factors, while lacking molecules essential for apoptosis or ECM remodeling such as cleaved-caspase 3,−8, and-9 (Yang et al., 2016). Indeed, upregulation of p53 expression has been reported in scarring phenotypes, a protein important for the inhibition of apoptosis (Tanaka et al., 2004).

Most lab-based *in vivo* assessment of wound closure and development is performed in rodents. This is mainly due to the high-throughput and low costs of these systems. However, it is important to understand that rodent wounds close differently to that of human's, primarily due to the process of contraction. This is mainly owed to an extensive subcutaneous striated muscle layer known as the panniculus carnosus that is virtually non-existent in humans. In rodents however, the panniculus carnosus allows the skin to move independently of the deeper muscles and is accountable for the rapid contraction of skin following injury. This physiological difference therefore creates difficulties to replicate the wound closure processes of human skin. This is a universal problem, one that is noted in much recent literature (Wang et al., 2013; Hu et al., 2018). Wang et al. discussed this problem, proposing an alternative model which involved splinting rodent wounds to inhibit contraction and force re-epithelization. Nevertheless, this model also encountered limitations including inflammation induced from sutures used to anchor the splint to the mouse skin which could influence any molecular changes (Dunn et al., 2013). Formerly published reports utilizing the splinted wound model lack descriptive details of splint management and exclusion criteria for removing animals from analysis in cases where splints might have been incompletely secured due to suture rupture or damage to the splint by the animal.

Another alternative method is the direct suturing of a scaffold to the edges of the experimental wounds. Anjum et al. conducted wounding experiments of this nature with (Nu/Nu) mice and found that contraction is still observed in all wounds, however a more reepithelialization route was observed in the central wound regions (Anjum et al., 2017). However, limitations of this method again point to the provoking of an inflammatory response and coincidently with an increased risk of surgical site infections (He et al., 2009). Suture knots, for example, can act as platforms for bacterial colonization and reproduction (Mashhadi and Loh, 2011).

To overcome these limitations, porcine models of wound healing are often used. Pigs are anatomically and physiologically similar to humans, and therefore can be considered excellent models of human diseases (Seaton et al., 2015; Acevedo et al., 2019). Indeed, the skin of pigs and humans are similar in that they have a relatively thick epidermis and dermal papillae (Montagna and Yun, 1964).

## Current Scar Treatments

There is a vast array of current treatments for scars which come in a variety of forms. Topical treatments such as Mederma® Skin Care gel (Merz Pharmaceuticals, Greensboro, NC, USA)^2^ is available over the counter. The active ingredients of Mederma® gel include onion extract; however, this product displayed no benefit when tested in a trial involving patients subjected to Mohs microsurgery (Jackson and Shelton, 1999).

Surgical revision is sometimes utilized for hypertrophic or normal scars. It is common practice in the clinic to wait several months before surgically excising scars, allowing them to become fully mature (Thomas and Somenek, 2012). The most direct excision technique for scar removal is surgical removal followed by linear closure of the skin. Surgery as a treatment, however, can result in excessive tension across the wound area or infection (Marshall et al., 2018). There are also many injectable treatments which can be used for scar treatment, including corticosteroids which is common therapy for keloid and hypertrophic scars (Thomas and Somenek, 2012).

A further example is Botulinum toxin [BOTOX® Cosmetic (onabotulinumtoxinA)^3^, Allergan, Irvine, CA], which is linked with improving scar appearance (Gassner et al., 2000). However, in a clinical trial in humans who presented with forehead wounds in an emergency department and were treated with botox or placebo, there was no difference in scar appearance in 3 of 4 visual scales upon suturing (Ziade et al., 2013).

Dressings are the traditional treatment mode for wound healing and scar reduction as a means of protecting the wounds, keeping a moist microenvironment, and offloading tension from the skin (Commander et al., 2016). Indeed, the use of simple paper tape alone has shown promise. When paper tape was applied to patients with cesarean section wounds for 12 weeks post-surgery, there was a reduction in scar formation, and it decreased the probability of the patient developing hypertrophic scars (Atkinson et al., 2005).

With current treatments offering varying degrees of efficacy it is imperative to develop novel modalities of treatment. The electrospinning of polymers holds potential in this regard.

## Electrospinning

**Figure 2:**
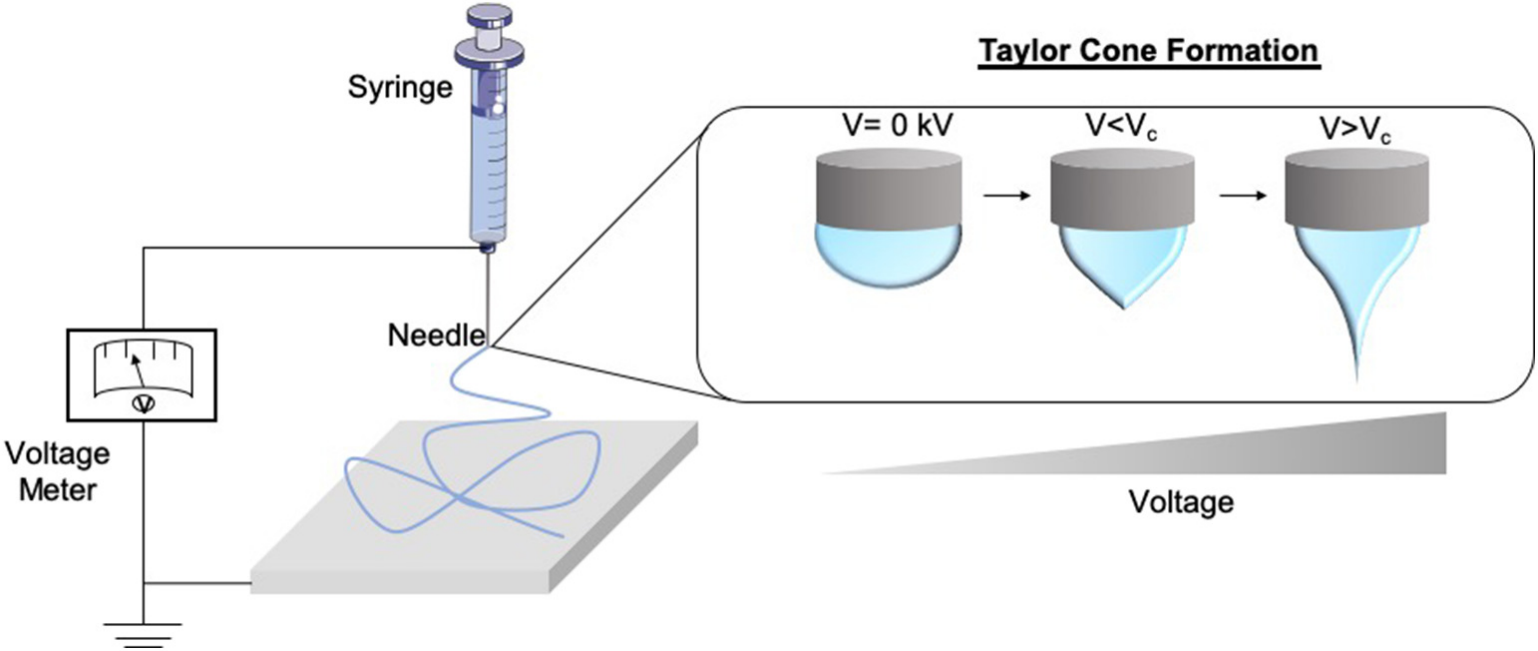
Schematic of the formation of the Taylor Cone. As the voltage supply to the electrospinning rig increases and surpasses the critical voltage (V_c_), the repulsion within the charged polymer overcomes its surface tension, fabricating solid nanofibers onto the collector. This is known as the Taylor Cone effect.

Electrospinning forms fibres through the application of an electrostatic field to a polymer solution (Cui et al., 2007; Bhardwaj and Kundu, 2010; Lagaron et al., 2017). The process of electrospinning can produce fibers right down to nanoscale and have applications in various fields, for example, wound dressings, drug delivery, and tissue engineering devices. The process itself is rapid and can be scaled to meet industrial demands to continuously produce fibers. This technique involves the use of a high voltage field strength from as low as 1 kV cm^1^, to charge the surface of a polymer solution droplet, subsequently inducing the ejection of a liquid jet toward a grounded surface. When the voltage reaches the optimal threshold [critical voltage (V_C_)] by radial charge repulsion, the single jet will divide into multiple filaments; this is recognized as the Taylor cone effect (Figure 2). This cone formation results in the construction of solidified fibers as the solvent evaporates. The V_C_ value varies between polymers due to alterations in chemical properties (Quinn et al., 2018). Solution properties such as viscosity, concentration, and dielectric constant, and operational parameters including the strength of the applied voltage, jet to collector distance, and flux, will all affect the morphology of the resulting fibers (Sencadas et al., 2012; Haider et al., 2015). Electrospun fibers possess high surface area to volume ratios, meaning they exhibit many of the desirable properties of an effective wound dressing such as protection from mechanical stimuli and providence of excellent gaseous exchange, and as such a lot of research has gone into optimizing them for this application. The following sections, therefore, explore the use of electrospun fibers in the treatment of scars and the potential future applications this technology could hold as a therapeutic (Table 1).

**Table 1:** Therapeutic potential of various electrospun polymers including therapy loaded nanofibers and tissue engineering options.

**Electrospun devices**	**Electrospun polymers**	**Incorporated therapeutics**	**Therapeutic outcomes**	**References**
Polymers 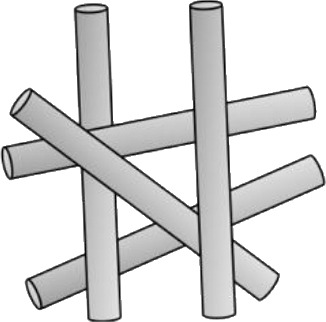	Recombinant Human Collagen, Chitosan and PEO	–	This dressing resulted in elevated levels of collagen III *in vivo* 14 days post-surgery in normal male SD rats compared to control groups, which is indicative of less scar tissue formation.	Deng et al., 2018
	Collagen Type I and PCL	-	Significantly reduced the area of scar tissue formation in back skin wounds of Sprague-Dawley rat compared to controls as determined via H&E staining.	Bonvallet et al., 2014
	Silk fibroin/gelatin	-	This dressing inhibited scar tissue formation *in vivo* via stimulating wound closure (*p* < 0.05), enhancing angiogenesis, and successfully refining collagen organization.	Shan et al., 2015
	Silk fibroin/PEO	-	This dressing stimulated rapid collagen production with a similar architecture to normal skin.	Ju et al., 2016
Polymers + therapeutic agents 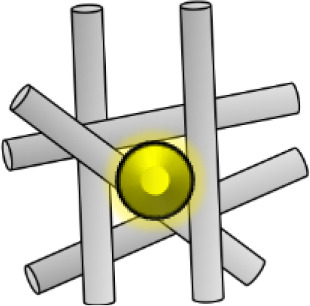	Collagen (Col, mimicking protein), PCL	Bioactive glass nanoparticles	This dressing delivered bioglass nanoparticles resulting in enhanced endothelial cell attachment and proliferation *in vitro*. This translated to well organized collagen deposition and skin appendages *in vivo* compared to controls without nanoparticles in specific pathogen-free male Sprague-Dawley rats.	Gao et al., 2017
	PELA and PEG	pbFGF and PEI	This dressing after 4 weeks resulted in fully differentiated epidemic cells, closely arranged basal cells and elevated occurrences of hair and sebum, consistent with the epithelial structure of normal skin.	Yang et al., 2012
	Poly(ε-caprolactone) (PCL)/gelatin	TGF-β1 inhibitor (SB-525334)	This dressing released PGT to effectively inhibit fibroblast proliferation *in vitro* and this translated to the successful prevention of hypertrophic scar formation *in vivo* in a full-thickness wound model on the ear of female New Zealand white rabbits.	Wang et al., 2017
Polymers + cells 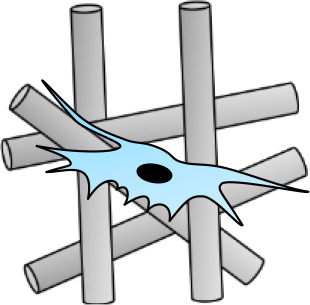	Recombinant Human Tropoelastin	Adipose derived stem cells (ADSC)	This dressing significantly improved wound closure and enhanced epithelial thickness *in vivo* in a murine excisional wound model compared to controls. It is hypothesized that the device could persist within the skin after healing and improve the overall tensile strength of the resulting scar tissue.	Machula et al., 2014
	Collagen and PCL	Keratinocytes and Fibroblasts	This layered dressing becoming unrecognizable after Day 21 post-implantation, indicative of high grafting efficiency.	Mahjour et al., 2015
	PLGA and Collagen	Bone marrow-derived MSCs (BM-MSCs)	This dressing resulted in faster wound closure times *in vivo* in full-thickness wound models in rats, with wounds closing 8 days earlier than controls.	Ma et al., 2011

**Figure 3:**
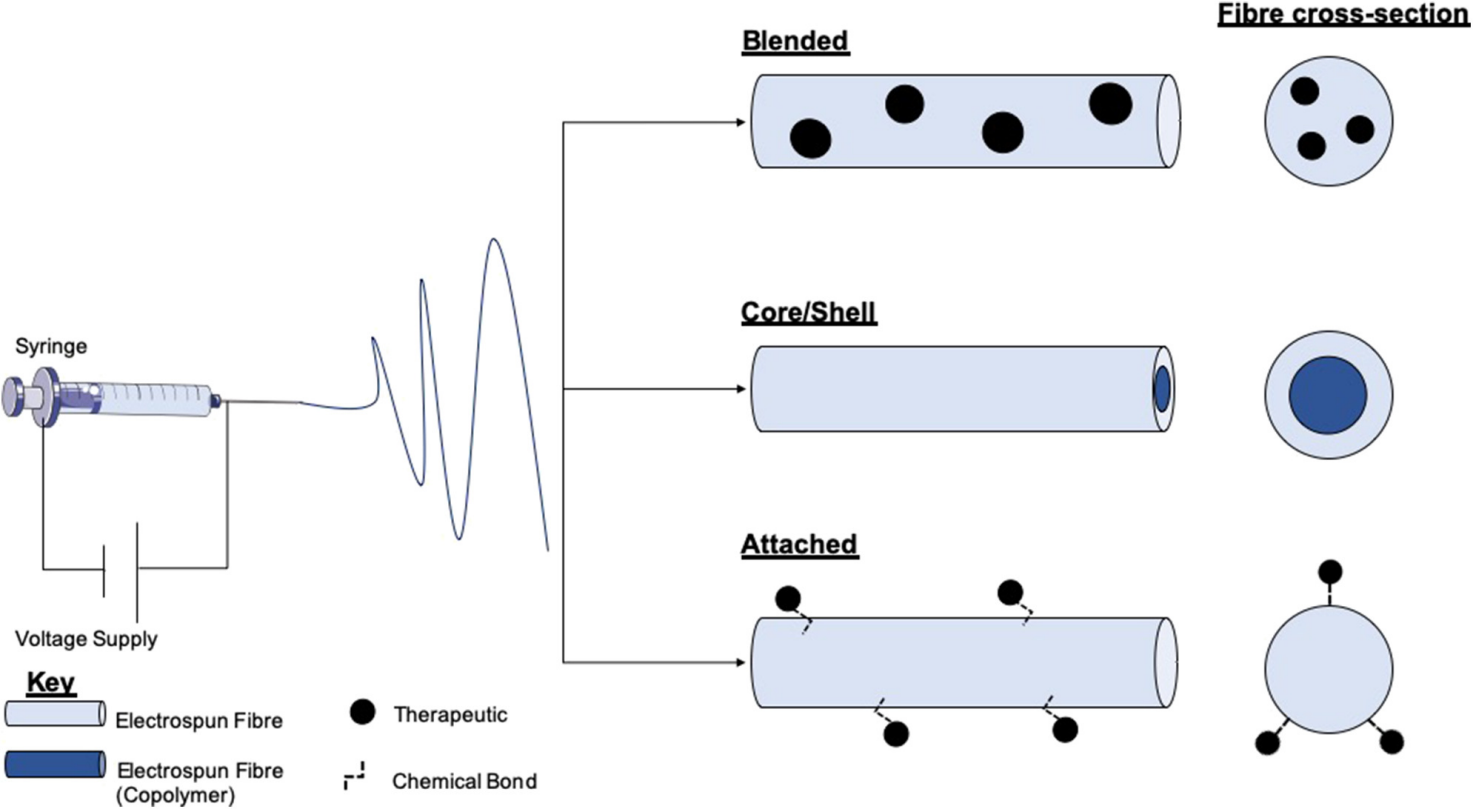
Schematic of electrospinning equipment and the resultant fibers and therapy loaded variants. The electrospinning set-up comprises a high voltage (V) supply which is connected to a syringe loaded with a polymer solution. Within the blending technique therapeutics or therapeutic precursors are mixed with polymer solutions before electrospinning. Either co-axial or emulsion electrospinning produces core/shell morphology fibers. This method allows for the encapsulation of therapeutics in either layer. Therapeutic attachment to fibers post-electrospinning allows for the loading of fragile molecules which cannot endure the electrospinning process.

Electrospinning is a versatile process which encompasses many different modes of fabrication for the incorporation of therapeutics (Figure 3). Blending is the predominant method for drug incorporation into electrospun nanofibers. The process of blending consists of drug or drug precursors encapsulated by means of dissolving or dispersing it into the polymer solution, before subsequent electrospinning. As the drug itself is in direct contact with the polymer, the drug-polymer interaction must be analyzed to ensure functionality is retained and that the drug can be adequately released from the product fibers. For example, Yang et al. investigated the use of gold nanoparticles modified with an antibacterial intermediate (6-aminopenicillanic acid) for wound healing applications. The gold nanoparticles were assimilated into electrospun nanofibers composed of polycaprolactone (PCL) and gelatin by blending. The nanoparticles release profile was investigated by nanofiber dipping into saline. The results showed that after Day 1 20.4% of the gold had been released, increasing to 65.7% by Day 7. Burst release was apparent within the initial days which can be attributed to a percentage of the gold existing near the surface of the fibers (Yang et al., 2017).

Modification of nanofiber surfaces to allow incorporation of therapeutics is another method for drug and cell loading (Prabhakaran et al., 2008; Ma et al., 2011; Wakuda et al., 2018). This method is advantageous for avoiding burst discharge of therapeutics and results in a more gradual release profile (Im et al., 2010). This technique is particularly beneficial for biomolecular therapeutics such as enzymes as surface conjugation, and slow-release helps to preserve functionality (Zamani et al., 2013). Plasma treatment of polymers is a typical method of surface modification. Nanofiber treatment with plasma in the presence of oxygen, ammonia, or air has resulted in the generation of amine or carboxyl groups on the surface of the fibers (Baker et al., 2006; Yan et al., 2013). This process functionalizes fibers for a variety of applications, such as the adhesion of the collagen or gelatin, which are key proteins found in the extracellular matrix, and so can improve cell adhesion and proliferation (He et al., 2005; Koh et al., 2008). It has been shown that poly(lactic-co-glycolic acid) PLGA nanofibers can be transformed to contain carboxylic acid groups through plasma glow discharge in the presence of oxygen and gaseous acrylic acid (Park et al., 2007). These fibers exhibited enhanced fibroblast cell adhesion and proliferation, desirable properties for wound healing.

The fabrication of core/shell nanofibers is another attractive method of bioactive incorporation into electrospun nanofibers. The production of electrospun core-shell nanofibers is accomplished through either co-axial or emulsion electrospinning. Co-axial electrospinning is a two-stream process that results in the fabrication of multipolymer fibers with the inner stream being the “core,” and the outer polymer passed stream forms the shell (Jiang et al., 2014). This method is auspicious for the incorporation of fragile cargos (e.g., DNA or growth factors) as the therapeutic interaction with the shell polymer blend which may be produced with harsh solvents is minimized, therefore preserving the cargo (Ghosh et al., 2008; Xie et al., 2016; Cheng et al., 2019). Wei et al. utilized this technique for the development of a wound dressing, comprising a PCL core and collagen shell nanofibers. The shell was blended with silver nanoparticles to take advantage of the anti-bacterial activity, and the core permeated with vitamin A, which has been shown to help with wound healing by increasing intra- and extracellular hydration (Campos et al., 1999; Wei et al., 2016). Emulsion electrospinning produces nanofibers of core/shell morphology by first introducing an emulsion into an initial polymer solution before the addition of a surfactant to isolate the different phases from each other (Liao et al., 2009). Castro et al. manufactured nanofibers composed of PCL and PCL/gelatin, which retained and delivered ketoprofen by solution and emulsion electrospinning, respectively. It was reported that using emulsion electrospinning of PCL/gelatin could diminish the burst release of ketoprofen compared with single PCL nanofibers, and sustained drug release for >100 h. Furthermore, the combination of gelatin into the nanofibers resulted in an increase in the cell proliferation of L929 fibroblast cells (murine) (Basar et al., 2017). It should be noted however that emulsion electrospinning can cause damage to molecules such as DNA via interface tension between the organic and aqueous phases within the emulsion (He et al., 2012).

## Electrospinning Polymers for Scar treatment

With the process of electrospinning being so versatile, a plethora of both synthetic and natural polymers can be processed to form fibrous structures with the potential to promote scar-free wound healing. Expectedly, not every polymer can be easily electrospun. Many factors influence this ability, including polymer viscosity, concentration, and entanglement. For polymers with inadequate characteristics for electrospinning, a copolymer can be employed to improve mechanical properties. Alginate is an example of a naturally occurring polymer with a well-noted history for improving wound healing due to its excellent ability to swell and maintain a moist microenvironment, which aids healing (Aderibigbe and Buyana, 2018). However, alginate alone does not possess ideal attributes for electrospinning due to its low chain entanglement (Nie et al., 2008). Poly (vinyl alcohol) (PVA) is a reputable polymer for electrospinning and as a result is frequently selected as a copolymer. Indeed, PVA is extensively used on an industrial scale and is favored in the medical industry due to excellent physical properties, processability, and biocompatibility. Tarun et al. developed an electrospun matrix composed of PVA/ sodium alginate (Tarun and Gobi, 2012). It was demonstrated that the matrix displayed excellent water vapor transmission rates, thus maintaining a moist wound microenvironment. Furthermore, *in vivo* studies using the full-thickness wound model in rats exhibited seemingly new epithelium development, void of any local adverse reactions. Indeed, the movement of epithelial cells across the surface of a wound is enabled in a wound that is kept moist, and in turn, promotes efficient healing (Field and Kerstein, 1994). Wounds that are kept moist typically present with less scar tissue formation (Atiyeh et al., 2003).

A further example of a natural polymer includes chitosan, which has noted antibacterial and antifungal properties, which would be highly beneficial for a wound dressing. It was suggested by Ignatova et al. that the crosslinking of PVA/Q-chitosan (a chitosan derivative) through the photo-crosslinking electrospinning procedure, would have antimicrobial effects on both Gram-positive and Gram-negative bacteria (Ignatova et al., 2006). The author's results showed that the matrix had exceptional resistance to the growth of bacteria exhibiting activity against *E. coli* and *S. aureus*. However, it is important to note that with polymers like chitosan there are drawbacks. Chitosan, as an example, is poorly soluble (Shete et al., 2012), and so tends to be dissolved in acidic conditions, namely using acetic acid or trifluoroacetic acid for example (Geng et al., 2005; Bazmandeh et al., 2019; Gu et al., 2019). The toxicity and cost associated with such solvents can imped the potential of chitosan in wound and anti-scarring therapies (Mengistu Lemma et al., 2016), however, during the electrospinning process much of the solvent evaporates under ideal conditions, and so could help alleviate these unwanted side effects when harsher solvents are required (Golecki et al., 2014; Haider et al., 2015).

Another natural polymer of noted potency as a wound-healing material is silk fibroin, a protein produced by some insects (e.g., silkworm). Fibroin makes for an excellent wound repair candidate as it is highly biocompatible, contains anti-inflammatory properties, and has notable anti-scarring potential. As such, much attention has turned to the electrospinning of silk to fabricate bioactive wound dressings. For example, Ju et al. developed electrospun silk fibroin nanofibers as a dressing material for the treatment of burn wounds. The authors found that the expression of IL-1α, which is pro-inflammatory, had significantly lower expression levels in silk fibroin treated skin compared to a gauze control treatment in the skin of male Sprague-Dawley rats where second degree burn wounds were induced on the backs. Further to this, the expression profile of TGF-β1 peaked at Day 21 post-wounding before declining, compared to at in gauze treated wounds which crested at Day 7. It was also noted that the silk fibroin nanofibers induced rapid collagen formation, which organized within the wound in a similar fashion to that of normal skin as opposed to a scarring composition (Ju et al., 2016).

## Electrospun Polymers With Added Therapeutics for Scar Treatment

With the variety of production avenues for electrospinning nanofibers (blending, core/shell, attachment), there lays the opportunity for the incorporation and delivery of a variety of anti-scaring therapeutics. As discussed, alginate offers an excellent polymer choice for wound dressings as it promotes a moist wound environment, and hence reduces the extent of scarring. In a study by Shalumon et al. the use of electrospun sodium alginate/PVA nanofibers loaded with ZnO nanoparticles (via the blending method) as an antibacterial wound dressing was explored. The study concluded that a concentration of between 0.5 and 5% is required for the fibers to have antibacterial activity as tested with *S. aureus* and *E. coli*, with minimal cytotoxic effects (using L929 murine fibroblast cells) (Shalumon et al., 2011). These nanofibers were tested *in vivo* using C57BL/6J mice, where UVB irradiation was employed to produce visible skin lesions, and scar formation was evident within 48–96 h. When these lesions where treated with the nanofiber dressings it was reported that no burn marks were detectible after 24 h post-injury. This rapid recovery was further confirmed by the downregulation of inflammatory cytokines IL-6, IL-1B, and TNF-a after 24 h compared to untreated controls. Taken together this electrospun device shows excellent potential for the reduction of scar tissue formation in a burn wound model (Hajiali et al., 2016).

A historic but still pertinent avenue for the treatment of wounds and scars is the use of essential oils (Sequeira et al., 2019). Previous research as developed electrospun nanofibers composed of alginate/polyethylene glycol (PEO) infused with lavender essential oil, for the treatment of UV-induced skin burns. These fibers showed antibacterial efficacy *in vitro* against *S. aureus*, and furthermore reduced the production of pro-inflammatory cytokines both *in vitro* and *in vivo* (Hajiali et al., 2016). The authors found that the burns of mice treated with the lavender-infused nanofibers healed faster compared to the untreated group. Karami et al. developed electrospun fibers of PCL and polylactic acid (PLA) which encapsulated thymol from thyme essential oil for the treatment of skin infections, with a focus on *E. coli* and *S. aureus* (Karami et al., 2013). Application of these nanofibers *in vivo* using a full-thickness wound model in Male Wistar rats resulted in an enhancement in granulation tissue formation and re-epithelialization at 14 days post-wounding (92.3%) compared to gauze (68%) and commercial (Comfeel Plus) (87%) controls. Histologically, the wounds treated with the commercial dressing exhibited some epidermal tissue at day 14, but this was more extensive in the nanofiber treated wounds (Karami et al., 2013). These results demonstrate the power of essential oils as efficient wound healing therapeutics, which could be employed for the reduction in scar tissue formation.

In another study conducted by Gao et al., endothelial progenitor cells were cultured on composite fibers consisting of PCL/collagen and bioactive glass nanoparticles *in vitro* (Gao et al., 2017). *In vivo* wound healing studies using specific pathogen-free male Sprague-Dawley rats revealed evident blood vessel formation, as well as upregulation of angiogenic markers such as hypoxia-inducible factor−1 alpha (HIF-1α) and vascular endothelial growth factor (VEGF). Throughout the *in vivo* study, wound healing potential was superior in wounds treated with nanofibers containing the bioglass nanoparticles. These bioglass-loaded nanofibers achieved 60% wound closure in the first week and ~90% closure with 2 weeks, compared to nanofibers containing no bioglass beads which achieved only 50 and 80% closure within week 1 and week 2, respectively. The total area of scar tissue in wounds treated with the bioactive glass-loaded nanofibers was significantly smaller and with highly organized collagen deposition compared to treatment with unloaded nanofibers (Gao et al., 2017).

**Figure 4:**
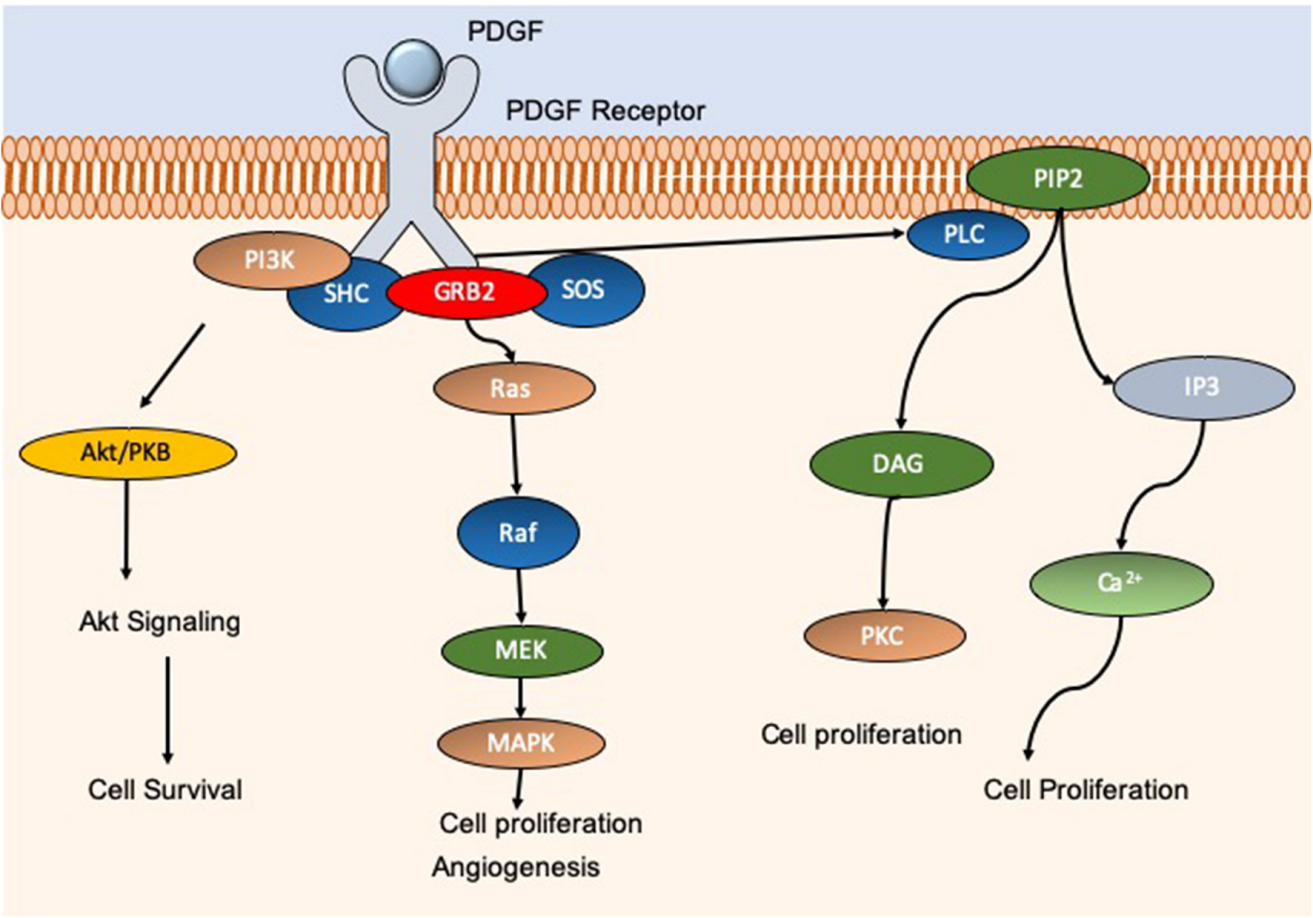
Schematic showing the molecular pathways associated with PDGF. PDGF activation gives rise to an increase in cell proliferation, migration, and angiogenesis through AkT, MAPK, and calcium pathways for example. PDGF is essential for efficient wound repair and minimization of potential scar tissue. Commercial product REGRANEX® utilizes recombinant human PDGF for the treatment of diabetic foot ulcers.

Many studies have explored the use of electrospun nanomaterials in the treatment of diabetic foot ulcers (DFU). DFU are categorized as a major complication of diabetes mellitus, and typically present on the feet, toes, and heels. DFU result from peripheral neuropathy, poor circulation, and impaired immune function, or a combination of these foundations. Of diabetic patients in the USA, 20% of foot ulcer cases displayed insufficiencies in peripheral atrial supply. Moreover, 50% of patients primarily displayed peripheral neuropathy, and ~30% presented with a combination of these conditions (Reiber et al., 1999)^4^ As such, with a wide-reaching cohort of diabetic patients suffering from chronic wounds, there is a continuous need for efficient wound healing options which result in rapid and scar-free results. Indeed, Yang et al. developed an electrospun dressing composed of poly(dl-lactide)-poly(ethylene glycol) (PDLLA-PEG) fibers which had polyethyleneimine (PEI)/pbFGF polyplexes incorporated by the emulsion loading technique, advantageous for the integration of fragile genetic material. PEG was also added to the shell portion of the fibers to aid in smooth release of the cargo. The authors found that the structure was able to sustain release of the polyplexes over a 4-week period, and successful transfection was observed that ensued for over 28 days, enhancing the proliferative capacity of the mouse embryo fibroblast cells *in vitro*. The efficacy of the fibers *in vivo* was tested in skin wounds generated on the dorsal area of diabetic male Sprague Dawley (SD) rats. The fibers containing PEI/pbFGF complexes resulted in a significantly higher wound recovery rate compared to untreated wounds, exhibiting improved vascularization and completed re-epithelialization (Yang et al., 2012). These are important outcomes as the more rapid and efficient cell migration corresponds to a reduction in scar tissue formation (Hadjizadeh et al., 2017). Yuan et al. also explored the combination of growth factors and electrospun materials for skin regeneration. The authors utilized a dual-spinneret electrospinner to manufacture fibers composed of chitosan- PEO and fibrinogen, loading the polymers with PDGF via blending directly before electrospinning (Yuan et al., 2018). PDGF is a critical player in wound repair initiation and progression, acting as a chemotactic instrument for neutrophils, monocytes, and fibroblasts (Sá et al., 2018). Furthermore, PDGF can block fibroblast differentiation into myofibroblasts thus decreasing scar formation (Yuan et al., 2018). Indeed, a therapy option of wound repair termed Regranex® is the only growth factor wound treatment for diabetic ulcers currently FDA approved, utilizing recombinant human PDGF (Figure 4) (Fang and Galiano, 2008). A single daily application of Regranex® has been shown to enhance wound closure, with a 30% faster healing time observed. It must also be noted that in the case of Regranex® it was previously suggested that it increased the rate of mortality related to malignancy development in patients treated with >3 tubes of the product, as concluded from a post-marketing retrospective study. However, this warning has since been removed from the product packaging, and many studies have disproved this theory (Ziyadeh et al., 2011; REGRANEX®, 2018). Nevertheless, daily application is considered to be inconvenient for the patient, impacting the quality of life. Thus, the delivery of this growth factor in a controlled manner could be of great benefit. In the instance of Yuan and authors, it was observed that the nanofibers developed exhibited an average fiber diameter of 202.3 ± 113.2 nm, PDGF integrity was retained, and upon release promoted an increase in the migration rate of human dermal fibroblasts. With regards to cytotoxicity, there was a significant decrease in the viability of cells exposed to the nanofibers (unloaded) at 72 h post-incubation compared to a no scaffold control. It is postulated that this decrease is the result of the degree of acetylation of chitosan, which has been shown to elicit strong cellular interactions due to positive charges (Aranaz et al., 2009). Indeed, other groups have observed a decrease in cell viability after 24 h in bladder carcinoma cells exposed to chitosan with a degree of acetylation greater than 50% (Younes et al., 2016). This is an interesting observation, and so should be factored into the rational for novel wound healing devices. However, it is suggested that this observation may be unique to *in vitro* experimentation, as prior studies using chitosan observed negligible toxic effect *in vivo*, linked with metabolic clearance of biodegradation products (Kean and Thanou, 2010; Jeong et al., 2017).

## Cell Delivery Via Electrospun Polymers for Scar treatment

The use of cells in regenerative medicine is widely explored and considered by many to hold great promise^5^. For example, mononuclear umbilical cord cells are easily available and have few associated ethical issues. Furthermore, it has been previously demonstrated that these cell types can be cultured on electrospun nanofibers (Chua et al., 2006).

In light of the advantageous potential of cell therapies for regenerative purposes, they are not without limitations. Therapies of this nature would require a large number of donors, these cells would not survive past low passage numbers, and there is the potential risk of rejection from the host (Venkat et al., 2018). A further example of potential cell therapies includes allogeneic bone marrow cells, which are easily available but carry the risk of rejection and would require specialized techniques for cell harvest and separation. Martinello et al. used allogenic mesenchymal stem cells to treat wounds in a large animal study using female Bergamasca sheep. It was found that at 15 Days post-injury, when compared to a control group, sheep treated with allogeneic bone marrow cells presented with a higher degree of wound closure, reepithelialization, as well as a reduction in inflammation. The latter of which the authors postulate may result in a decreased myofibroblast development and thus scar formation (Martinello et al., 2018).

In saying this, autologous cells helps mitigate the problem of immune rejection, but nevertheless require the same specialized harvesting and separation expertise (Venkat et al., 2018; Ramotowski et al., 2019).

Many studies have explored the use of cells for wound healing and scar tissue minimization. However, the most typical route for this exploration is the direct injection of cells, which can be highly inefficient, and incurs substantial cell death due to shear forces through the injection needle (Burdick et al., 2016). Electrospun nanofibers may be the solution for the efficient and effective delivery of cells for skin regeneration. Electrospun nanofibers have high surface areas, and therefore have the capacity to retain many cultured cells (Chen et al., 2009). For example, it was shown by Zhao et al. that electrospun nanofibers composed of silk fibroin could successfully host cardiomyocytes (Zhao et al., 2019). This capability of electrospun fibers to host cells is thought by many to be owed to the likeness to the natural ECM, fitting to the fibrous nature of collagen, thereby facilitating the natural proliferation of cells (Ramakrishna et al., 2006).

The disadvantage of using these nanofibers for this purpose, however, is the restricted control over pore structure. The pore size in this instance is proportional to the fiber diameters, with smaller diameters resulting in smaller pore sizes, which consequently can decrease cell infiltration (Wu and Hong, 2016). In some cases, cells only infiltrate the uppermost portion of the nanofibers, which reduces the advantages of three-dimensional cell culture. When nanofibers are compared to microfibers in this regard, it has been shown that larger pore sizes, inherent to macrofibers, promote stem cell differentiation, coupled with improved cellular permeation, however nanofibers are associated with higher cell attachment (Wu and Hong, 2016).

Mahjour et al. developed a skin substitute composed of electrospun PCL/collagen fibers for the treatment of burn wounds. The fibers in question were layered in a composite manner, with different layers infused with keratinocytes (top layer) and fibroblasts (bottom layer). The electrospun fibers were applied in an *in vivo* mouse model of wound healing. Data collected from the study showed that the cell incorporated composite fibers integrated into the wound bed in a highly effective way, becoming unrecognizable after Day 21 post-implantation. This was in comparison to blank fiber scaffolds which received the lowest score of integration. Furthermore, at Day 21, wounds treated with the cell-incorporated composite fibers had 7% remaining non-reepithelialized skin and 45% wound contraction, whereas the blank composite fibers had only 21% re-epithelialization and 56% wound contraction (Mahjour et al., 2015).

Mesenchymal stem cells (MSCs) are multipotent stem cells typically isolated from bone marrow, adipose tissues, and the dermis (Orbay et al., 2012). MSCs have shown avid potential in skin repair, promoting angiogenesis, reducing inflammation, and facilitating the establishment of an ECM (Jackson et al., 2012). When MSCs enter an inflammatory environment a switch to an immunomodulatory phenotype is initiated by Interferon-gamma (IFNγ), Tumor Necrosis Factor-alpha (TNFα) and IL-1β (Ren et al., 2008). When this phenotype is active; there is evidence suggesting that MSCs can suppress the proliferation of B cells as well as natural killer cells (Corcione et al., 2006; Sotiropoulou et al., 2006). This suppression enhances the acute immune response to damage and can reduce a pro-fibrotic response that can result from sustained inflammation (Redd et al., 2004). Williams et al. tried to reduce scar sizes in ischemic cardiomyopathy through injection of allogeneic MSCs (Williams et al., 2013). The authors suggested that MSCs could reverse ventricular remodeling, and, indeed, it was shown that MSCs stimulate endogenous cardiac stem cells to proliferate and differentiate. The resulting mature cardiomyocytes exhibited therapeutic effect by secretion of growth factors and cytokines.

Similarly, Li et al. showed that MSCs loaded into a 3D graphene foam decreased scar tissue formation. The foam resulted in upregulation of VEGF as well as bFGF leading to enhanced neovascularization, as well as heightening levels of TGF-β3, which prevents scarring. The MSC loaded foams were tested *in vivo* in a full-thickness wound model using wild-type rats. The use of MSCs in the foam resulted in a significant closure of the wound from day 3 post-wounding compared to controls of untreated and unloaded foam, this trend was observed consistently until endpoint at 14 Days post-wounding (Li et al., 2015).

With such obvious potential, it is not surprising that many researchers have explored the incorporation of stem cells like MSCs into electrospun nanofibers for wound and scar treatments. For example, Ma et al. integrated bone marrow-derived MSCs (BM-MSCs) into nanofibers comprised of collagen and PLGA. The device demonstrated enhanced healing profiles in a full-thickness wound model *in vivo* using rats. The wounds treated with the BM-MSCs loaded nanofibers resulted in faster closure times compared to the untreated control, closing 8 days earlier (Ma et al., 2011). Furthermore, it was observed that localized treatment with the BM-MSCs resulted in a decrease in myofibroblast numbers. The authors postulated this may be due to MSCs ability to express hepatocyte growth factor 8 which inhibits myofibroblastic differentiation (Ma et al., 2011). These results suggest that this device would reduce scar tissue formation whilst allowing rapid and efficient wound repair.

In another study, Machula et al. electrospun nanofiber membranes of tropoelastin seeded with adipose-derived stem cells for wound healing applications (Machula et al., 2014). The authors found that the stem cells rapidly proliferated on the nanofibers and partook in efficient ECM establishment, covering the entire scaffold *in vitro*. Application of the cell-nanofiber device in an *in vivo* excisional wound model with female SCID mice showed an enhancement in wound closure and restoration of normal epithelium compared to control wounds treated with petrolatum jelly–impregnated gauze. The average thickness of re-epithelized skin tissue for the control and stem cell-nanofiber treated groups was 27.7 ± 7.8 μm and 51.9 ± 11.27 μm, respectively (*p* = 0.001). It is postulated by the authors that the electrospun tropoelastin device may persist within the scar tissue of healed skin and in doing so enhance the tensile strength of any resultant scar tissue.

A limitation associated with stem cell culture on electrospun nanofibers is that small pore size may result in a blockage in nutrient diffusion and cellular infiltration. If this problem can be mitigated, it could lead to a highly potent regenerative device.

## Conclusions and Future Directions

As detailed in this review, the use of electrospun nanofibers for scar treatment has substantial potential. With a plethora of polymers being “*electrospinnable*” alone or in conjunction with therapeutic agents or cells, the problem of scar management could be significantly improved. However, much work is still required to get such therapies into the clinic. Indeed, there are currently 5 clinical trials exploring the use of electrospun nanofibers^6^; yet, none of these are scar tissue-specific, and no current trials are recruiting. Conversely, there are 760 clinical trials listed for skin scarring^7^, 140 of which are recruiting^8^. This suggests that much research is focusing on novel therapies for scar treatment and prevention, which is a favorable scenario, and indeed these therapies if approved could in future be incorporated into electrospun nanofibers. Recent results have been notably discouraging. Metelimumab, for example, a TGF-β1 targeting antibody, exhibited no improvement in the treatment of systemic sclerosis compared to a placebo control^9^. In saying this, an RNAi-based inhibitor of connective tissue growth factor (CTGF) termed RXI 109 has completed phase I trials and is now in phase II trials for hypertrophic scar treatment^10^. With promising therapies in the pipeline, it is exciting to hypothesize the efficiency of their delivery via electrospun nanofibers.

Encouragingly, electrospun nanofibers can be manufactured on an industrial scale, with the production of continuous nanofibers from a variety of polymers already proven (Ramachandran and Gouma, 2008; Zhang et al., 2012; Ma et al., 2015; Wang et al., 2018). Translating nanofiber production from laboratory to commercial scale is readily accommodated through the application of multi-jet nozzle electrospinners, which have been reported to process as much as 6.5 kg/h of polymer to produce fibers (Persano et al., 2013). Current commercial examples include the Zeus Bioweb™ composites, composed of electrospun polytetrafluoroethylene (PTFE). The Bioweb™ exhibits a high surface area and possesses an advantageously minute pore size, typically in the range of 1–4 μm. Zeus boasts a variety of Bioweb™ applications including scaffold potential and implantable structures in the body^11^. A further commercial example of an electrospinning product is the SpinCare™ system by Nicast. SpinCare™ is a handheld device that fabricates nanofibers from polymers directly for tailored wound healing applications. These nanofibers provide a semi-permeable coverage facilitating excellent moisture regulation. The fibers are also comfortable as they are made to fit the shape of the patient's wound^12^.

It is well-believed that the combination of gene therapies with biomaterials hold great potential as future generation therapeutic devices (Bleiziffer et al., 2007; Goker et al., 2019). Although promising, gene therapy is not without limitations. For example, therapies of this nature are historically challenging to deliver into cells, due to similarities in charges between nucleic acids and cell membranes. Viral gene delivery is the most common form of gene therapy due to its high efficiency. These vectors, however, are met with numerous trepidations as they can result in mutagenesis and have a restricted capacity for genetic material (Mingozzi and High, 2013). Non-viral options for gene therapy also exist and include cationic polymers (Olden et al., 2018), liposomes (Balazs and Godbey, 2011), and peptides (McCarthy et al., 2014; Cole et al., 2019). Indeed, recent literature published by Mulholland et al. explored the delivery of an siRNA complexed with a cell penetrating peptide termed RALA from an electrospun bilayer wound patch. The use of the RALA peptide significantly enhanced the transfection efficient of the nucleic acids *in vitro* using HMEC-1 endothelial cells, downregulating expression anti-angiogenic FK506-binding protein-like FKBPL. This high efficiency translated to significant upsurge in angiogenic activity *in vivo* in wounds on the backs of C57BL/6 mice, resulting in an increase in blood vessel density of 326% compared to untreated wounds (Mulholland et al., 2019). This technology holds excellent potential for scar tissue treatment as a vast array of nucleic acids could be delivered in this manner.

Taken together, with the literature and the state-of-the-art technology discussed in this review, it can be rationalized that electrospinning shows great promise for the development of next generation devices for the treatment and management of scars.

## Author's Note

This manuscript was an invited paper for the article collection on Biomaterials for Skin Wound Repair: Tissue Engineering, Guided Regeneration, and Wound Scarring Prevention.

## Author Contributions

EM wrote the manuscript and produced all figures and tables with the aid of SMART Servier Medical Art www.smart.servier.com (Attribution 3.0 Unported (CC BY 3.0).

## Conflict of Interest

The author declares that the research was conducted in the absence of any commercial or financial relationships that could be construed as a potential conflict of interest.

## Footnotes

^1^https://www.prnewswire.com/news-releases/global-scar-treatment-market-2013-2018–2023—rise-in-online-retailing-of-scar-treatment-products-300698219.html

^2^https://www.mederma.com/

^3^https://hcp.botoxcosmetic.com/Contact

^4^https://www.niddk.nih.gov/about-niddk/strategic-plans-reports/diabetes-in-america-2nd-edition

^5^https://www.mayoclinic.org/tests-procedures/bone-marrow-transplant/in-depth/stem-cells/art-20048117

^6^https://clinicaltrials.gov/ct2/results?cond=&term=electrospinning&cntry=&state=&city=&dist=&Search=Search (Date visited November 15, 2019).

^7^https://clinicaltrials.gov/ct2/results?cond=Skin+Scarring&term=&cntry=&state=&city=&dist= (Date visited November 15, 2019).

^8^https://clinicaltrials.gov/ct2/results?cond=Skin+Scarring&Search=Apply&recrs=a&age_v=&gndr=&type=&rslt= (Date visited November 15, 2019).

^9^https://clinicaltrials.gov/ct2/show/NCT00432328

^10^https://clinicaltrials.gov/ct2/show/NCT02246465

^11^https://www.zeusinc.com/products/biomaterials/bioweb-composites

^12^http://nicast.com/spincare/product-description/
